# Antitumor activity of zoledronic acid in primary breast cancer cells determined by the ATP tumor chemosensitivity assay

**DOI:** 10.1186/1471-2407-12-308

**Published:** 2012-07-23

**Authors:** Tanja Fehm, Manfred Zwirner, Diethelm Wallwiener, Harald Seeger, Hans Neubauer

**Affiliations:** 1 Department of Obstetrics and Gynecology, University of Tübingen, Calwerstr 7/6, 72076, Tübingen, Germany

## Abstract

**Background:**

The NeoAzure study has demonstrated that the use of the bisphosphonate zoledronic acid (Zol) in the neoadjuvant setting increases the rate of complete response in primary breast cancer and therefore indicates direct antitumor activity. The purpose of this study was to compare the antitumor effect of Zol with standard chemotherapy in primary breast cancer cells using ATP-tumor chemosensitivity assay (ATP-TCA).

**Methods:**

Breast cancer specimens were obtained from patients with breast cancer who underwent primary breast cancer surgery at the Department of Obstetrics and Gynecology, Tübingen, Germany, between 2006 through 2009. Antitumor effects of Zol, TAC (Docetaxel, Adriamycin, Cyclophosphamide) and FEC (5-Fluorouracil, Epirubicin, Cyclophosphamide) were tested in 116 fresh human primary breast cancer specimens using ATP-TCA. ATP-TCA results were analyzed with different cut-off levels for the half maximal inhibitory concentration (IC50), for IC90 and for the sensitivity index (IndexSUM). Each single agent or combination was tested at six doubling dilutions from 6.25, 12.5, 25, 50, 100, and 200% of test drug concentrations (TDC) derived from the plasma peak concentrations determined by pharmacokinetic data. The assay was carried out in duplicate wells with positive and negative controls.

**Results:**

The median IndexSUM value was lower for Zol than for the combined regimen FEC (36.8%) and TAC (12.9%), respectively, indicating increased antitumor activity of Zol in primary breast cancer cells. The difference regarding Zol and FEC was significant (p < 0.05). The median IC50 value for Zol (8.03% TDC) was significantly lower than the IC50 values for FEC (33.5% TDC) and TAC (19.3% TDC) treatment (p < 0.05). However, the median IC90 value for Zol (152.5% TDC) was significantly higher than the IC90 value obtained with TAC (49.5% TDC; p < 0.05), but similar to the IC90 value for FEC (180.9% TDC). In addition a significant positive correlation was observed for the IndexSum of Zol and the ER status (p < 0.01).

**Conclusion:**

Zoledronic acid has a strong antitumor effect on primary breast cancer cells *in vitro* which is equal or superior to commonly used chemotherapeutic regimens for treating breast cancer.

## Background

Bisphosphonates are standard of care in the treatment of metastatic bone disease in women with breast cancer and are also used to prevent and treat therapy-induced osteoporosis in the adjuvant setting [1-7]. These compounds significantly reduce bone pain and hypercalcaemia, maintain and improve quality of life while being generally well tolerated. Treatment with bisphosphonates should be initiated after the first diagnosis of bone metastases and should be continued life long based on the current recommendations treatment (also under disease progression) [8].

There is growing evidence that bisphosponates may have a direct antitumoric effect beyond their beneficial effect on bone metastasis. Various investigations have demonstrated that Zol can inhibit cell proliferation *in vitro*, can trigger apoptosis and inhibit neovascularization [9-11]. Similar effects have also been described for other bisphosphonates. Several studies have demonstrated a beneficial effect of the bisphosphonates clodronate and Zol in preventing disease progression in the adjuvant setting in early breast cancer patients and to improve disease free survival and overall survival as well [12-14].

The NeoAzure study has demonstrated that the use of the bisphosphonate Zol in the neoadjuvant setting increases the rate of complete response in primary breast cancer and therefore indicates direct antitumor activity [15].

The purpose of the present study was to compare the antitumor effect of Zol with standard chemotherapy in primary breast cancer cells using ATP-TCA.

## Results

In Table 1 the clinicpathological data for the tumor samples are presented. Tumor stages T1 and T2-4 were nearly equally distributed. Five percent of the tumors were of G1, about 60% were of G2 and about 36% were of G3 grade. Nearly 80% of the samples were positive for ER and PR expression, about 30% were HER2/neu positive. Most of the tumors were from node-negative patients.

**Table 1:** Histological data of the tumor samples (n = 116)

**Parameter**	**N (%)**
Tumor size	
pT1	52/115 (45.2)
pT2-4	63/115 (54.8)
Nodal status	
N0	72/111 (64.9)
N1	21/111 (18.9)
N2	10/111 (9.0)
N3	8/111 (7.2)
Metastasis	
M0	109/113 (96.4)
M1	4/113 (3.6)
Grading	
G1	6/112 (5.3)
G2	65/112 (58.0)
G3	41/112 (36.7)
Estrogen receptor	
pos	93/112 (83.0)
neg	23/112 (17.0)
Progesterone receptor	
pos	88/112 (78.5)
neg	28/112 (21.5)
HER2	
pos	36/114 (31.5)
neg	78/114 (68.5)

**Figure 1:**
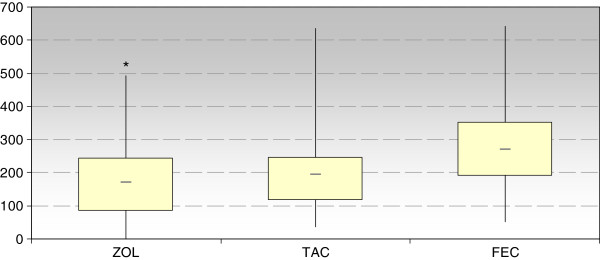
Median values of IndexSum (25 and 75% quantiles; min, max)) of zoledronic acid (Zol) in comparison to FEC and TAC (* p < 0.05 vs. FEC).

As shown in Figure 1 the median IndexSUM value was 36.8% and 12.9% lower for Zol than for the combined regimen FEC and TAC, respectively, indicating increased antitumor activity of Zol in primary breast cancer cells. The difference regarding Zol and FEC was significant (p < 0.05). No significant difference was found between Zol and TAC and between FEC and TAC.

Table 2 depicts that the median IC50 value for Zol (8.03% TDC) was significantly lower than the IC50 values for FEC (33.5% TDC) and TAC (19.3% TDC) treatment (p < 0.05). However, the median IC90 value for Zol (152.5% TDC) was significantly higher than the IC90 value obtained with TAC (49.5% TDC; p < 0.05), but similar to the IC90 value for FEC (180.9% TDC).

**Table 2:** IC90 and IC50 values (median %TDC; 25 and 75 quantiles) of zoledronic acid (Zol) in comparison to FEC and TAC

**Compound**	**IC 90**	**IC 50**
Zol	152.5 (36.3/217.8)^**+**^	8.0 (4.6/18.3)*
FEC	180.9 (101.7/229.7)	33.5 (11.9/75.9)
TAC	49.5 (39.4/86.8)	19.3 (7.3/29.2)

+ p < 0.05 vs. TAC; * p < 0.05 vs. FEC and TAC.

A significant positive correlation was found for the IndexSum of Zol and the ER status (n = 112; slope = 6.33; 95%CI = 2.13 to 10.5; p = 0.0035; Y = 130.8 + 6.33X; r^2^ = 0.07).

## Discussion

The first bisphosphonate was synthesized in 1865 [16]. Bisphosphonates are a further development of the pyrophosphates which are well-known in the chemical industry. However, in contrast to pyrophosphates they are not inactivated *in vivo*. In 1968, it was shown that bisphosphonates effectively inhibit osteoclast-mediated bone resorption and since then they have become an integral part in the management of benign and cancer-induced bone disease [17].

A wealth of preclinical studies demonstrates that bisphosphonates exhibit direct antitumor effects. *In vitro* studies have clearly shown that bisphosphonates, in particular Zol, induce tumour cell apoptosis and inhibit tumour cell adhesion, invasion and proliferation and angiogenesis [9,18,19].

Up to now the antiproliferative and proapoptotic effect of Zol was mainly proven in human breast cancer cell lines. For the first time we have investigated the *ex vivo* effect of Zol in freshly resected human breast specimen and compared it to the effect of commonly used chemotherapeutic regimen, i.e. FEC and TAC. The results revealed that Zol was at least equal or even superior in its antitumoric effect when compared to both combined regimens. The significance of the found correlation between the IndexSums obtained with FEC, TAC and Zol with T-, N- and ER-status of the tumors remains currently unknown.

Since our experiments have been performed with fresh breast tumor samples which provide only limited quantity of cells after disaggregation we were not able to apply alternative test systems such as MTT or XTT assay to validate the outcome. However, we have tested three breast cancer cell lines in response to the cytotoxic effect of Zol using the ATP-TCA with similar results.

The effector mechanisms of Zol have been investigated by several groups. Their data point towards reversing effects of Zol on cell proliferation and upregulation of pro-apoptotic combined with downregulation of anti-apoptotic proteins and survival factors [19]. The mevalonate pathway which is a target of Zol may be important for these observations [20].

These and our results indicate that Zol and probably all bisphosphonates may exert a direct antitumoric effect on breast cancer cells beyond their antimetastatic effect on bone matrix. Thus the use of bisphosphonate treatment as adjuvant therapy in women with breast cancer may have clinical significance.

This hypothesis is supported by some animal and clinical studies investigating the use of Zol beyond the action on cancer-induced bone loss. In a mouse model Zol demonstrated a significant inhibition of breast cancer tumor growth [17]. Further *in vivo* studies revealed synergistic antiproliferative effects of Zol in combination with doxorubicin [20], docetaxel [21,22] as well as paclitaxel [23].

Intermittent addition of Zol can significantly reduce serum levels of vascular endothelial growth factor (VEGF) as compared to basal levels [24].

In ABCSG-12 the addition of Zol to endocrine therapy was associated with a significant 36% improvement in disease free survival (DFS) compared with endocrine treatment alone, the median follow-up was 48 months [25]. Furthermore, antitumour benefits of zoledronic acid were observed outside bone.

Data from several pilot studies show that Zol potentially clears disseminated tumour cells (DTCs) from the bone marrow of patients with early breast cancer [26-28] or in the neoadjuvant setting with locally advanced breast cancer [29]. The presence of DTCs in the bone marrow is a recognized risk factor for the subsequent development of bone metastases, but longer term data are required to establish the clinical relevance of these findings.

The first subgroup analysis (n = 205) of the AZURE-study (Does Adjuvant Zoledronic Acid RedUce Recurrence in Breast Cancer) of patients with neoadjuvant chemotherapy revealed a synergistic effect of the combination of chemotherapy with Zol on primary tumor size [15]. The parameter *residual invasive tumor size* (RITS) was significantly reduced by 43% (CT alone 27.4 mm vs. CT + Zol 15.5 mm; p = 0,006) and *pathological complete response rate* improved by 69% (CT alone 6.9% vs. CT + Zol 11.7%; p = 0.146), which, however, was not statistically significant.

The mechanism(s) by which Zol may trigger a direct antitumoric action is currently not known. It inhibits farnesyl diphosphate synthase within the mevalonate pathway and, through this mechanism, is a potent inhibitor of osteoclast-mediated bone resorption. The mevalonate pathway is known to be probably involved in cancer development.

Our data for the first time suggest a positive correlation between the efficacy of Zol and the ER status. A possible association between the efficacy of Zol and the presence of ER on inhibition of tumor cell growth could be shown in two *in vitro* experiments that revealed that ER-negative cell lines are inhibited in their growth by Zol worse than ER-positive cell lines [10,11]. However, in other experimental studies the efficacy of Zol was similar in ER-negative and ER-positive cell lines [20] or even more pronounced in the ER-negative cell line [30].

Interestingly, in the *Women’s Health Initiative trial on bisphosphonate use and breast cancer incidence* a significant lower incidence of about 30% of ER-positive breast cancers was found in bisphosphonate users than in nonusers [31]. The mechanism(s) of a possible dependence of Zol from ER status remains unknown so far. Hints might be derived from prostate cancer, since in the prostate a dysregulated cellular growth is mediated by inhibiting the rate-limiting pathway step in cholesterol synthesis, thereby decreasing isoprenylate intermediates, decreasing cholesterol rich cellular membrane domains, and down-regulating androgen and estrogen receptors [32]. We therefore speculate that expression of ER might point towards high activity of cholesterol synthesis pathway and therefore high effect of blocking the mevalonate pathway by Zol. Further research is necessary to reveal this topic in more detail.

The Zol concentrations used in the present study were in the range of 0.625 to 20 μg/ml and the IC50 value was 1 μg/ml. The physiological plasma concentrations (Cmax) after infusion of Zol are in the range of 0.2 to 0.4 μg/ml within 24 h [33]. Thus the effective in vitro Zol concentrations were comparable to in vivo plasma concentrations.

## Conclusions

In summary zoledronic acid appears to elicit a strong antitumor effect on primary breast cancer cells *in vitro* which is equal or superior to commonly used chemotherapeutic regimens for treating breast cancer.

## Methods

### Tumor specimens

116 fresh human primary breast cancer specimens obtained from patients who underwent surgery at the Department of Obstetrics and Gynecology, Tübingen, Germany, between 2006 through 2010, were included in the study. The ATP-TCA was performed as a routine procedure immediately after surgery using the remaining part of the primary tumor specimen not needed for histopathology. All patients provided their informed consent and the study design was approved by the local Ethics Committee.

### In vitro ATP-TCA

Chemosensitivity was assessed using the ATP-TCA kit (TCA-100; DCS Innovative Diagnostik Systeme, Hamburg, Germany). The ATP-TCA has been previously described in detail elsewhere [34]. Briefly, surgical biopsies (1-2 cm^3^) were taken during primary surgery. Tumor cells were isolated by mechanical dissociation and enzymatic treatment with Collagenase Worthington Typ CLS III (Biochrom, Berlin, Germany) to obtain a single cell suspension. Dissociated epithelial cells were counted and assessed for viability using trypan-blue exclusion. A minimum of 7,500 viable cells was determined as the cut-off requirement and were then seeded into each well of a 96-well polypropylene microplate. Cells were incubated for 5 days at 37 °C/5% CO_2_ in complete assay medium (DCS Innovative Diagnostik Systeme) and treated with different test drug concentrations (TDC) in duplicates at six different doses. Two controls were included in each plate: a no drug control consisting of media only (M0) and a maximum inhibitor (MI) control which kills all cells present. At the end of the 5 day-incubation period, remaining cells were lysed by the addition of tumor cell extraction reagent (DCS Innovative Diagnostik Systeme). An aliquot of the lysate from each well was added to corresponding wells in a white 96-well microplate (Thermo Life Sciences, Basingstoke, UK) followed by addition of luciferin-luciferase reagent. The light output corresponding to the level of ATP present was measured using a luminometer (Berthold, Hamburg, Germany) and analyzed with custom software to provide both numerical and graphical results. Luminescence measurements are directly related to ATP levels and enable to determine the percentage of growth inhibition by reference to the control wells included with each plate.

### Drugs

Zol, TAC (docetaxel, adriamycin, cyclophosphamide) and FEC (5-fluorouracil, epirubicin, cyclophosphamide) were used in this study. Each single agent or combination was tested at six doubling dilutions from 6.25, 12.5, 25, 50, 100, and 200% of TDC derived from the plasma peak concentrations determined by pharmacokinetic and clinical information (10). Standard 100% TDC values were 1.29 μM for Zol, 17.3 μM for 5-fluorouracil, 0.088 μM for epirubicin, 1.24 μM for docetaxel and 1.11 μM for cyclophosphamide.

### Data analysis

ATP-TCA results were interpreted and compared by using the following calculation methods: (a) *IC90 and IC50*: comparing the drug concentrations that achieve 90% or 50% growth inhibition *in vitro*, which is calculated by interpolation (7) and (b) *a sensitivity index (IndexSUM)*: calculated by summing the percentage of tumor growth inhibition (TGI) at each concentration tested: IndexSUM =600− sum of % TGI at 200, 100, 50, 25, 12.5, and 6.25% TDC (11). The total inhibition of growth results in an *IndexSUM* of 0 and no inhibition of growth at any concentrations provides an *IndexSUM* of 600. The Wilcoxon-Rank test was used to compare the different regimen. A *p*-value of <0.05 was considered to indicate statistical significance. *Ex vivo* test results obtained with the tumor specimen were tested for correlation with tumor data according to the TNM and receptor status using regression analysis. All statistics were calculated with JMP 3.2.1 (SAS Institute, Cary, NC, USA).

### Immunohistochemical technique

Expression of estrogen and progesterone receptors (ER and PR) and HER2/neu was determined as descibed previously [35].

## Competing interests

The authors declare that they have no competing interests.

## Authors’ contributions

HN and HS participated in the design of the study and performed the assays and the statistical analysis. TF, MZ and DW conceived of the study, and participated in its design and coordination and helped to draft the manuscript. All authors read and approved the final manuscript.

## Pre-publication history

The pre-publication history for this paper can be accessed here:

http://www.biomedcentral.com/1471-2407/12/308/prepub
